# Inhibitors of Haemopoietic Cell Proliferation?: Specificity of Action Within the Haemopoietic System

**DOI:** 10.1038/bjc.1974.53

**Published:** 1974-02

**Authors:** B. I. Lord, L. Cercek, B. Cercek, G. P. Shah, T. M. Dexter, L. G. Lajtha

## Abstract

The specificity of action of mature blood cell extracts on their own progenitor cells was investigated by measuring their effects on the structuredness of the cytoplasmic matrix (SCM) using the technique of fluorescence polarization. Changes in SCM induced by the various extracts are probably closely related to the proliferation state of the cells.

Saline extracts of lymphocytes, granulocytes and erythrocytes (LNE, GCE and RCE respectively) have been partially purified by ultrafiltration into selected molecular weight ranges and each tested against proliferative populations of lymphoid, granulocytic and erythroid cells. In all cases, complete specificity of effect on SCM was found, LNEs affecting only lymphoid cell populations, GCEs affecting only the granulocytic cell populations and RCEs affecting only erythroid cells. In each case, with the possible exception of the RCEs, the active fractions reside in the molecular weight ranges reported in the literature for cell extracts possessing proliferation inhibitory properties.


*Br. J. Cancer* (1974) **29**, 168

### INHIBITORS OF HAEMOPOIETIC CELL PROLIFERATION?: SPECIFICITY OF ACTION WITHIN THE HAEMOPOIETIC SYSTEM

B. I. LORD, L. CERCEK, B. CERCEK, G. P. SHAH, T. M. DEXTER AND L. G. LAJTHA

*From the Paterson Laboratories, Christie Hospital and Holt Radium Institute, Manchester M20 9BX*

Received 19 November 1973. Accepted 22 November 1973

**Summary.—The specificity of action of mature blood cell extracts on their own progenitor cells was investigated by measuring their effects on the structuredness of the cytoplasmic matrix (SCM) using the technique of fluorescence polarization. Changes in SCM induced by the various extracts are probably closely related to the proliferation state of the cells.**

**Saline extracts of lymphocytes, granulocytes and erythrocytes (LNE, GCE and RCE respectively) have been partially purified by ultrafiltration into selected molecular weight ranges and each tested against proliferative populations of lymphoid, granulocytic and erythroid cells. In all cases, complete specificity of effect on SCM was found, LNEs affecting only lymphoid cell populations, GCEs affecting only the granulocytic cell populations and RCEs affecting only erythroid cells. In each case, with the possible exception of the RCEs, the active fractions reside in the molecular weight ranges reported in the literature for cell extracts possessing proliferation inhibitory properties.**

A NUMBER of investigators have claimed that extracts from the mature elements of the blood system (erythrocytes, granulocytes and lymphocytes) have specific inhibitory effects on the proliferation of the progenitors of those cells (Rytömaa and Kiviniemi, 1968; Moorhead *et al.*, 1969; Houck, Irausquin and Leikin, 1971; Kivilaasko and Rytömaa, 1971). These extracts have frequently been called " chalones " and a number of properties which a compound must demonstrate before qualifying for that title have been defined (NCI Monograph 38, 1973). One of these properties, that of specificity of action for cells of their own kind, has so far proved defiant to unequivocal demonstration. Most observations have depended on the uptake of tritium labelled thymidine into cells synthesizing DNA in culture systems; systems which can very rapidly deteriorate unless exact culture conditions are maintained throughout. Comparisons have been made of the inhibition of thymidine uptake by one cell type with that of other cell types in the culture. However, it is well recognized that while granulocytic cells can be maintained in a reasonably healthy condition under various culture conditions, erythroid cells are difficult to culture.

Consequently, comparative measurements of these two types of cells in the same culture system are rather unreliable and can give only an indication of the direction of an effect.

The use of thymidine as a marker leads, in itself, to problems. It has been demonstrated that tissue extracts often contain thymidine. This serves to dilute the specific activity of the labelled thymidine, which then can result in lowered incorporation, *i.e.* apparent inhibition of DNA synthesis. Lenfant, Kren-Proschek and Verly (1973) demonstrated this effect, for example, in their work on the liver " chalone ".

Another difficulty arising from reports of work undertaken to demonstrate cellular specificity is that comparative tests are often made using similar extracts from widely differing tissues, *e.g.* muscle, liver, brain, kidney, fibroblasts, lung, skin, Harding–Passey melanoma (Bullough and Laurence, 1970; Houck and Irausquin, 1973; Rytömaa, 1973) and testing them on haemopoietic tissues. Alternatively, the haemopoietic extracts have been tested on other types of tissue, *e.g.* fibroblasts (Houck, Weil and Sharma, 1972). In many cases, the end points in comparative tests may be different, *e.g.* cellularity, tritiated thymidine incorporation or colony production. Erythroid, granulocytic and, in the mouse at least, lymphoid cells develop from a common haemopoietic stem cell and are very closely related. It is therefore, necessary, in order to demonstrate absolute specificity of action, to test all the extracts on the progenitor cells of each haemopoietic cell line.

In this paper a new method of approaching the problem of specificity of growth control will be described, in which it has proved possible to compare the effects of various blood cell extracts on each type of haemopoietic cell line, under virtually identical conditions and with the same end point. To make these comparisons, the structuredness of the cytoplasmic matrix (SCM) was measured with the technique of fluorescence polarization elaborated by Cercek and Cercek (1972*a*, 1973*c*). The technique is based on the excitation of fluorescein molecules, produced by enzymatic hydrolysis of the non-fluorescing fluoresceindiacetate (FDA) in the cytoplasm with polarized light and, measurement of the polarization of the emitted fluorescence. Rotational relaxation of the fluorescein molecule between absorption and emission of light depolarizes the fluorescence. Rotation of the fluorescein molecules depends on the physical state of organization of the cytoplasmic matrix at the molecular level (Cercek and Cercek, 1973*a*). This organization is the result of physical interactions between macromolecules such as proteins, water molecules and solutes (Ling, 1972). Perturbations of these interactions result in a change in SCM (Cercek and Cercek, 1972*a*, *b*, 1973*a*, *b*; Cercek, Cercek and Ockey, 1973*c*; Cercek, Cercek and Garrett, 1973*d*). The degree of fluorescence polarization (P) increases with increasing SCM and *vice versa*.

### MATERIALS AND METHODS

### *Preparation of test extracts*

(a) *Lymph node extracts* (LNE).—100 g fresh bovine mesenteric lymph nodes were stripped of fat from their capsules and minced in a Waring blender for 30 seconds in physiological saline. The freed cells and debris from the nodes were spun down and the supernatant discarded. The pellet was resuspended in 1 l saline and incubated overnight at 4°C. After incubation, the cellular material was spun down (20 min at 2500 *g*) and the supernatant retained as the crude extract. This was dialized against distilled water (2 changes at a volume ratio of approximately 20 : 1) for 48 hours at 4°C to precipitate euglobulins, which were then removed by centrifugation. The supernatant was filtered through Whatman No. 2 filter paper to remove fat globules and then through a series of millipore filters of reducing pore size, finishing with a 0·22 μm filter for sterilization. Finally, it was fractionated by ultrafiltration through Amicon Diaflo filter membranes to obtain a partially purified extract in the molecular weight range 30,000–50,000 daltons. According to published data, the active lymphoid inhibitor lies in this range (Lasalvia, Garcia-Giralt and Macieiro-Coelho, 1970; Houck *et al.*, 1971).

For comparison with the erythrocyte and granulocyte extracts (see below), further Diaflo fractions were obtained in the molecular weight range of 500–1000 daltons and 1000–10,000 daltons. In these cases, however, the dialysis step was omitted.

(b) *Granulocyte extracts* (GCE).—Thirty male Wister rats, 250–300 g body weight were injected i.p. with 2 doses of 15 ml of 3·5% polyvinylpyrrolidone, 17 hours apart. 2–4 hours after the second injection, peritoneal fluid was removed from the rats and 250 i.u. of heparin added to the fluid to prevent coagulation. The cells were washed in saline and then resuspended in fresh saline at a concentration of $5 \times 10^6$ cells/ml. Each rat yielded approximately $2 \times 10^8$ cells, of which about 85% were mature granulocytes and 15% were macrophages. The cell suspension was then incubated for 2 hours at 37°C and the cells removed by centrifugation; the supernatant was passed through millipore filters and finally fractionated on Diaflo filters. The molecular weight range fractions retained were 500–1000 daltons, 1000–10,000 daltons and, for comparison with the LNEs, 30,000–50,000 daltons. The 2 smaller fractions include the molecular weight reported both for the active GCE and for the active erythroid cell inhibitor (Rytömaa and Kiviniemi, 1968; Paukovits, 1971; Kivilaasko and Rytömaa, 1971).

(c) *Erythrocyte extracts* (RCE).—Ten male Wistar rats, 250–300 g body weight were bled from the axillary vessels under ether anaesthesia; 100 i.u. of heparin was added to prevent coagulation. Red blood cells were sedimented by centrifugation (10 min at 500 *g*) and the plasma and buffy coat removed. The erythrocytes were washed twice in saline and finally resuspended in saline at about 50% haematocrit. Extracts were then prepared and fractionated as from the granulocytes.

*Preparation of test cells*

Human peripheral lymphocytes were prepared from blood collected in Searle-LH/10 lithium heparin containing vials. Macrophages were removed by the carbonyl iron technique (Kuper, Bignall and Luckcock, 1961). Lymphocytes in a pure state (> 90%) were obtained by the Ficoll–Triosil gradient separation (Harris and Ukaejiofo, 1969). The lymphocytes were washed twice with saline and once in TC Medium 199 (Wellcome Ltd) and resuspended in TC Medium 199 at the concentration of $5 \times 10^6$ cells/ml. For stimulation of lymphocytes a 5-times diluted Reagent Grade PHA (Wellcome Ltd) was used; 0·1 ml of the diluted PHA were added to 1 ml of cell suspensions. This stimulated lymphocyte culture served as the baseline control for the subsequent inhibitor testing.

Granulocytic cells proliferating in a long term (2–10 week) culture system developed by Dexter *et al.* (1974) were harvested, washed and resuspended in serum-free TC 199 medium. In such a culture at least 90% of the cells are granulocytic and in all stages of development.

Normoblasts from mouse foetal liver were used for the third cell population. Livers were removed from 16-day old embryos, broken into a single cell suspension in TC 199 medium by aspiration through progressively smaller needles, washed and resuspended in serum-free TC 199 medium; 90 to 95% of the nucleated cells in this suspension are developing erythroid cells.

*Measurement of SCM*

SCM was measured by the degree of fluorescence polarization of fluorescein molecules in the cytoplasmic matrix. For this purpose the cells were suspended at concentrations of $10^5$ cells/ml in 2·5 $\mu$mol/l fluoresceindiacetate (FDA) solution in phosphate buffered saline. The suspension was rapidly transferred into a 1 cm cuvette and put into the thermostatically controlled cuvette holder of the Perkin–Elmer MPF-2A fluorescence spectrophotometer fitted with the polarization accessory. Measurements were made at 27°C. The excitation monochromator was set at 473 nm and the emission monochromator at 510 nm. Details of the experimental procedures and calculations of fluorescence polarization values, P, were the same as those described in experiments when changes in SCM were measured in Chinese hamster ovary cells (Cercek *et al.*, 1973*c*). We estimate the values of P in these experiments to be accurate to within 5%.

For the purpose of measurements all test cell suspensions were reconstituted to a concentration of $5 \times 10^6$ cells/ml. In the case of lymphocytes an initial measurement of the SCM was made before stimulating with PHA. A further measurement was made 15–30 min after PHA and this served as the control value for subsequent tests. The control SCM value for the other 2 cell types was measured on the initial cell suspension. Each test extract was dissolved in TC 199 medium at a concentration of 100 $\mu$g/ml and added to an aliquot of the test cell suspension to a final concentration of 33 $\mu$g/ml. All three extracts and fractions (500–1000, 1000–10,000 and 30,000–50,000 daltons) were tested on all 3 cell preparations. In all cases replicate measurements were made at 30 and 40 min after treating with the test extract. At the end of each series of tests repeat measurements of the controls were made to check that the original condition of the cells had not changed.

### RESULTS

Three major series of experiments were carried out. First, lymphocytes prepared in the manner described were tested with all 9 fractions. In each case, the SCM of the cells was measured 30–40 min after treating the PHA stimulated lymphocytes with the appropriate fractions of the extracts. The results are shown in Table I. Normal lymphocytes of the particular donor had a fluorescence polarization value, P, (SCM) of 0·191 which on stimulation with PHA decreased to 0·142 (74% of normal). Since normal lymphocytes are non-proliferating, the SCM value of PHA stimulated lymphocytes was taken as the 100% level for comparison with extract treated samples. It can be seen from the Table that the only extract to have an effect was the 30,000–50,000 dalton extract from lymph nodes, the P value rising to 0·205, 144% of the stimulated control. All other extracts were without effect.

TABLE I.—*SCM of Normal Human Peripheral Lymphocytes*

| Treatment | | Polarization value P | P % control |
|---|---|---|---|
| Normal lymphocytes (NL) | | 0·191 | 135 |
| NL+PHA (30 min) | | 0·142 | 100 |
| Extract | Molecular weight range | | |
| Lymph node | 500–1000 | 0·145 | 102 |
| extract | 1000–10000 | 0·153 | 107 |
| (LNE) | 30000–50000 | 0·205 | 144 |
| Granulocyte | 500–1000 | 0·144 | 101 |
| extract | 1000–10000 | 0·145 | 102 |
| (GCE) | 30000–50000 | 0·142 | 100 |
| Erythrocyte | 500–1000 | 0·140 | 99 |
| extract | 1000–10000 | 0·140 | 99 |
| (RCE) | 30000–50000 | 0·140 | 99 |

The SCM of cells is measured by the fluorescence polarization value, P. All measurements were made 30–40 min after treatment with cell extract.

With the cultured granulocytopoietic cells (Table II) the only extract to have a significant effect on the initial SCM (P = 0·176) was the 500–1000 daltons fraction from granulocytes. This extract increased the SCM to a value of P = 0·249 (142% of control). The larger GCE fraction at 1000–10,000 daltons was also somewhat effective, raising the SCM to P = 0·199 (113% of control). The high molecular weight fraction of GCE, the LNE fractions and the RCE fractions were all without effect.

TABLE II.—*SCM of Granulocytopoietic Cells*

| Treatment | | Polarization value P | P % control |
|---|---|---|---|
| Granulocytopoietic cells | | 0·176 | 100 |
| Extract | Molecular weight range | | |
| Lymph node | 500–1000 | 0·183 | 104 |
| extract | 1000–10000 | 0·171 | 98 |
| (LNE) | 30000–50000 | 0·180 | 102 |
| Granulocyte | 500–1000 | 0·249 | 142 |
| extract | 1000–10000 | 0·199 | 113 |
| (GCE) | 30000–50000 | 0·183 | 104 |
| Erythrocyte | 500–1000 | 0·171 | 98 |
| extract | 1000–10000 | 0·171 | 98 |
| (RCE) | 30000–50000 | 0·168 | 96 |

The SCM of cells is measured by the fluorescence polarization value, P. All measurements were made 30–40 min after treatment with cell extract.

In the same way for the erythroid cells, the initial SCM of P = 0·147 of foetal liver normoblasts (Table III) was changed only by the extracts from erythrocytes. However, in this case, all 3 RCEs were equally effective, the 500–1000, 1000–10,000 and 30,000–50,000 dalton fractions respectively raising the SCM to values of P = 0·223 (151% of control), 0·217 (148% of control) and 0·215 (146% of control).

TABLE III.—*SCM of Foetal Liver Normoblasts*

| Treatment | | Polarization value P | P % control |
|---|---|---|---|
| 16d. Foetal liver cells | | 0·147 | 100 |
| Extract | Molecular weight range | | |
| Lymph node | 500–1000 | 0·149 | 102 |
| extract | 1000–10000 | 0·155 | 105 |
| (LNE) | 30000–50000 | 0·150 | 102 |
| Granulocyte | 500–1000 | 0·142 | 97 |
| extract | 1000–10000 | 0·153 | 104 |
| (GCE) | 30000–50000 | 0·152 | 104 |
| Erythrocyte | 500–1000 | 0·223 | 151 |
| extract | 1000–10000 | 0·217 | 148 |
| (RCE) | 30000–50000 | 0·215 | 146 |

The SCM of cells is measured by the fluorescence polarization value, P. All measurements were made 30–40 min after treatment with cell extract.

A more limited experiment was also carried out on proliferating lymphocytes. Normal peripheral lymphocytes were stimulated with PHA. After 2 hours the cells were washed and the culture re-established without PHA for a further 46 hours. The dose of PHA was sufficient to allow the subsequent proliferation of the lymphocytes (Sörén, 1973) which at 48 hours were at the first main peak of DNA synthesis. These cells were washed, re-suspended in serum-free TC 199 medium and treated with LNE (30,000–50,000 daltons) and GCE (500–10,000 daltons). Table IV shows the result. From the SCM value of P = 0·156 of cultured lymphocytes, LNE increased the SCM to P = 0·291 (186% of control) but the GCE was without effect (P = 0·153 or 98% of control).

TABLE IV.—*SCM of Cultured Human Peripheral Lymphocytes*

| Treatment | Polarization value P | P % control |
|---|---|---|
| Cultured lymphocytes | 0·156 | 100 |
| add lymph node extract 30,000–50,000 daltons fraction | 0·291 | 186 |
| add granulocyte extract 500–1000 daltons fraction | 0·153 | 98 |

The SCM of cells is measured by the fluorescence polarization value, P.

### DISCUSSION

Progression of cells from the resting phase ($G_0$ or $G_1$) into the cell cycle is accompanied by a decrease in SCM. This biophysical change affects the rate constants of enzyme reactions in the cell and it is therefore to be expected that changes in SCM could be important in the regulation of proliferative processes (Cercek and Cercek, 1973*a*; Cercek, Cercek and Ockey, 1973*c*).

Triggering of normal human lymphocytes into cell cycle by mitogens such as phytohaemagglutinin, results in an immediate and with time progressive decrease in SCM (Cercek, Cercek and Garrett, 1973*d*). By analogy, we make the hypothesis that the biological effects of growth inhibitors such as " chalones " will be accompanied by an increase in SCM, inducing a biophysical state which is characteristic for the cells in, or approaching, the resting phase of the cells.

To ascertain that the observed increase in the degree of fluorescence polarization, P, are indeed the result of changes in SCM due to the biological effects of inhibitors and not a physicochemical phenomenon of selective quenching of the fluorescein fluorescence by the extracts, the yields of fluorescein fluorescence in the presence and absence of extracts have been measured. The extracts used in our experiments had no effect on the quantum yield, indicating that the increases in values of P are caused by changes in SCM.

With regard to the experimental results, Table V is a composite of Tables I–III and shows the percentage change in SCM in each of the tests. The extracts considered to be active in inhibitory function are shown in heavy type. For LNE, this is 30,000–50,000 daltons (Lasalvia *et al.*, 1970; Houck *et al.*, 1971). For both GCE and RCE it is reported as < 5000 daltons, probably 2000–4000 daltons (Rytömaa and Kiviniemi, 1968; Paukovits, 1971; Kivilaasko and Rytömaa, 1971). However, Dr D. J. Pillinger of these laboratories suggested, on the basis of Sephadex column fractionation, that the real activity may in fact reside somewhere below 1000 daltons (personal communication). Accordingly, both the 500–1000 and 1000–10,000 dalton fractions are shown in heavy type as possible inhibitory regions. It is clear from this Table that there is a highly significant correlation between the " active " extracts and the cell types which are affected. A complete picture of specificity of blood cell extracts for cells of their own type is demonstrated.

The exception to correlating the effect completely with the supposedly " active " fractions is the large (30,000–50,000 daltons) red cell extract which is as effective as the 2 smaller ones. This may

TABLE V.—*Percentage Change in SCM of Haemopoietic Cells when Treated with Mature Blood Cell Extracts*

| Test extract | | Cell type tested | | |
|---|---|---|---|---|
| Extract | Molecular weight range | Normal lymphocytes | Cultured granulocytic cells | Foetal liver normoblasts |
| Lymph node extract (LNE) | 500–1000 | +2 | +4 | +2 |
| | 1000–10000 | +7 | −2 | +5 |
| | **30000–50000** | **+44** | +2 | +2 |
| Granulocyte extract (GCE) | **500–1000** | +1 | **+42** | −3 |
| | **1000–10000** | +1 | **+13** | +4 |
| | 30000–50000 | 0 | +4 | +4 |
| Erythrocyte extract (RCE) | **500–1000** | −1 | −2 | **+51** |
| | **1000–10000** | −1 | −2 | **+48** |
| | 30000–50000 | −1 | −4 | **+46** |

Molecular weight ranges shown in heavy type are the ranges containing active inhibitory material according to the reported data (see text). Highly significant changes in the SCM of the test cells are also shown in heavy type.

be due to a feature of the fractionation technique. Each fraction is necessarily contaminated to a small degree by the lower molecular weight fractions since the method depends simply on differential molecular concentration. In each ultrafiltration procedure a small volume of concentrate is retained which has not passed through the filter. The degree of contamination depends on the initial volume of crude extract. In the case of red cell extracts this was low so that contamination of the 30,000–50,000 dalton fraction with the smaller molecular sizes was relatively high. However, none of the LNE nor GCE fractions have any effect on the foetal liver cells so that the specificity of RCE for normoblasts remains beyond question. The same argument may be applied to the GCE. Contamination here is smaller because the initial volume of crude extract is higher. The relatively small effect of the 1000–10,000 dalton fraction may simply be due to such a contamination therefore, and add weight to Dr Pillinger's suggestion that inhibitory activity is likely to reside in the $<$ 1000 dalton molecular weight range.

In these comparisons the lymphoid test cells are probably not, strictly speaking, proliferating. PHA initiates the processes leading to a cell cycle but whether the cells are by this stage, *i.e.* 15–30 min after application of PHA to the lymphocytes in the $G_1$ phase of a cell cycle is doubtful. The effect of the large LNE fraction on these cells is, however, clearcut but the question arises whether in this case it can be related to proliferation inhibition. By allowing the PHA-lymphocyte culture to proceed for 48 hours before testing, the lymphocytes certainly are in cell cycle and, as Table IV shows, LNE produced a profound effect on the SCM of these cells whereas the granulocyte active GCE fraction remained ineffective. Further studies in these laboratories have shown that LNE inhibits the incorporation of tritiated thymidine by PHA stimulated lymphocytes irrespective of when it is applied throughout the whole 72 hours period of a PHA culture (Shah and Lord, unpublished observations).

Clearly the problem associated with possible thymidine contamination of the extract affecting the incorporation of labelled thymidine does not enter into this sort of measurement. However, it is possible that thymidine may affect the proliferation of the cells directly. Accordingly, measurements of SCM of PHA stimulated lymphocytes were made after treatment with thymidine solutions. Solutions of thymidine at 0·3 $\mu$g/ml (equivalent to 1% contamination of the extract with thymidine) and at 3·3 $\mu$g/ml (equi-

TABLE VI.—*Effect of Thymidine on SCM of Normal Human Peripheral Lymphocytes*

| Treatment | Polarization value P | P % control |
|---|---|---|
| Normal lymphocytes (NL) | 0·196 | 100 |
| NL+PHA | 0·145 | 74 |
| 0·3 μg thymidine per ml (NL+PHA) | 0·150 | 76·5 |
| 3·3 μg thymidine per ml (NL+PHA) | 0·138 | 70·5 |

The SCM of cells is measured by the fluorescence polarization value, P.

valent to 10% contamination) were incubated for 30 min with the stimulated lymphocytes. Table VI shows the results. It is clear that these high concentrations of thymidine ( $> 10^{-2}$ mmol/l compared with, for example, $< 10^{-3}$ mmol/l concentration of active LNE) have no direct effect on the SCM of lymphocytes. It is, however, barely conceivable that the degree of specificity demonstrated by the results shown in this paper could be accounted for by the thymidine contamination.

The questions of dose of extract, the timing of the application of the extract and the nature of the effect are still to be elucidated, but from these preliminary observations it is clear that extracts from the mature cellular elements of haemopoietic tissues are capable of producing an effect which is specific for the precursor cells of their own kind only. While the measurement made, that of changes of SCM, may be due to changes other than in the proliferative state of the cells, it is clear that changes in the cell's proliferative state do produce definitive changes of SCM. It is probable, therefore, that the extracts may be having a " chalone-like " effect, *i.e.* that of inhibiting proliferation of the cells.

It is also worth noting that the lymphoid extracts were of bovine origin; the granulocyte and erythrocyte extracts were from rats. By contrast, the test lymphoid cells were human and the granulocytic and erythroid cells were from mice. Thus, all these tests were successful across the species barrier, which is another of the criteria quoted for the " chalone " type compounds.

This work was supported by grants from the Cancer Research Campaign and the Medical Research Council.

### REFERENCES

BULLOUGH, W. S. & LAURENCE, E. B. (1970) The Lymphocyte Chalone and its Antimitotic Action on a Mouse Lymphoma *in vitro*. *Eur. J. Cancer*, **6**, 525.

CERCEK, L. & CERCEK, B. (1972*a*) Studies on the Structuredness of Cytoplasm and Rates of Enzymatic Hydrolysis in Growing Yeast Cells. I. Changes Induced by Ionizing Radiation. *Int. J. Radiat. Biol.*, **21**, 445.

CERCEK, L. & CERCEK, B. (1972*b*) Studies on the Structuredness of Cytoplasm and Rates of Enzymatic Hydrolysis in Growing Yeast Cells. II. Changes Induced by Ultra-violet Light. *Int. J. Radiat. Biol.*, **22**, 539.

CERCEK, L. & CERCEK, B. (1973*a*) Relationship between Changes in the Structuredness of Cytoplasm and Rate Constants for the Hydrolysis of FDA *Saccharomyces cerevisiae*. *Biophysik*, **9**, 109.

CERCEK, L. & CERCEK, B. (1973*b*) Effect of Centrifugal Forces on the Structuredness of Cytoplasm in Growing Yeast Cells. *Biophysik*, **9**, 105.

CERCEK, L., CERCEK, B. & OCKEY, C. H. (1973*c*) Structuredness of the Cytoplasmic Matrix and Michaelis-Menten Constants for the Hydrolysis of FDA during the Cell Cycle in Chinese Hamster Ovary Cells. *Biophysik*, **10**, 187.

CERCEK, L., CERCEK, B. & GARRETT, J. V. (1973*d*) Biophysical Differentiation between Normal Human and Chronic Lymphocytic Leukemia Lymphocytes. Proc. 8th Leukocyte Culture Conference, Uppsala. New York, London: Academic Press. p. 553.

DEXTER, T. M., ALLEN, T. D., LAJTHA, L. G., SCHOFIELD, R. & LORD, B. I. (1973) Stimulation of Differentiation and Proliferation of Haemopoietic Cells *in vitro*. *J. cell Physiol.*, **82**, 461.

HARRIS, R. & UKAEJIOFO, E. O. (1969) Rapid Preparation of Lymphocytes for Tissue-typing. *Lancet*, ii, 327.

HOUCK, J. C., IRAUSQUIN, H. & LEIKIN, S. (1971) Lymphocyte DNA Synthesis Inhibition. *Science, N.Y.*, **173**, 1139.

HOUCK, J. C., WEIL, R. L. & SHARMA, V. K. (1972) Evidence for a Fibroblast Chalone. *Nature, New Biol.*, **240**, 210.

HOUCK, J. C. & IRAUSQUIN, H. (1973) Some Properties of the Lymphocyte Chalone. In *Chalones: Concepts and Current Researches*. Ed. B. K. Forscher and J. C. Houck. *N.C.I. Monog.*, **38**, 117.

KIVILAASKO, E. & RYTÖMAA, T. (1971) Erythrocyte Chalone, a Tissue Specific Inhibitor of Cell proliferation in the Erythron. *Cell Tiss. Kinet.*, **4**, 1.

KUPER, S. W. A., BIGNALL, J. R. & LUCKCOCK, E. D. (1961) A Quantitative Method for Studying Tumour Cells in Blood. *Lancet*, i, 852.

LASALVIA, E., GARCIA-GIRALT, E. & MACIEIRA-COELHO, A. (1970) Extraction of an Inhibitor of DNA Synthesis from Human Peripheral Blood Lymphocytes and Bovine Spleen. *Rev. Eur. etud. clin. Biol.*, **15**, 789.

LENFANT, M., KREN-PROSCHEK, L. & VERLY, W. G. (1973) Thymidine as One of the Factors in a Beef Liver Extract Decreasing Tritiated Thymidine Incorporation into DNA. *Can. J. Biochem.*, **51**, 654.

LING, G. N. (1972) Hydration of Macromolecules. In *Water and Aqueous Solutions: Structure, Thermodynamics and Transport Processes*. Ed. R. A. Horne. New York, London: Wiley Interscience. p. 692.

MOORHEAD, J. F., PARASKOVA-TCHERNOZEMSKA, E., PIRRIE, A. J. & HAYES, C. (1969) Lymphoid Inhibitors of Human Lymphocyte DNA Synthesis and Mitosis *in vitro*. *Nature, Lond.*, **224**, 1207.

N.C.I. Monograph, **38**, 1973.

PAUKOVITS, W. R. (1971) Control of Granulocyte Production: Separation and Chemical Identification of a Specific Inhibitor (Chalone). *Cell Tiss. Kinet.*, **4**, 539.

RYTÖMAA, T. (1973) Chalone of the Granulocyte System. In *Chalones: Concepts and Current Researches*. Ed. B. K. Forscher and J. C. Houck. *N.C.I. Monog.*, **38**, 143.

RYTÖMAA, T. & KIVINIEMI, K. (1968) Control of Granulocyte Production. I. Chalone and Antichalone, two Specific Humoral Regulators. *Cell Tiss. Kinet.*, **1**, 329.

SÖRÉN, L. (1973) Effects of Removing PHA from Cultures of PHA-stimulated Lymphocytes. *Expl cell Res.*, **79**, 350.


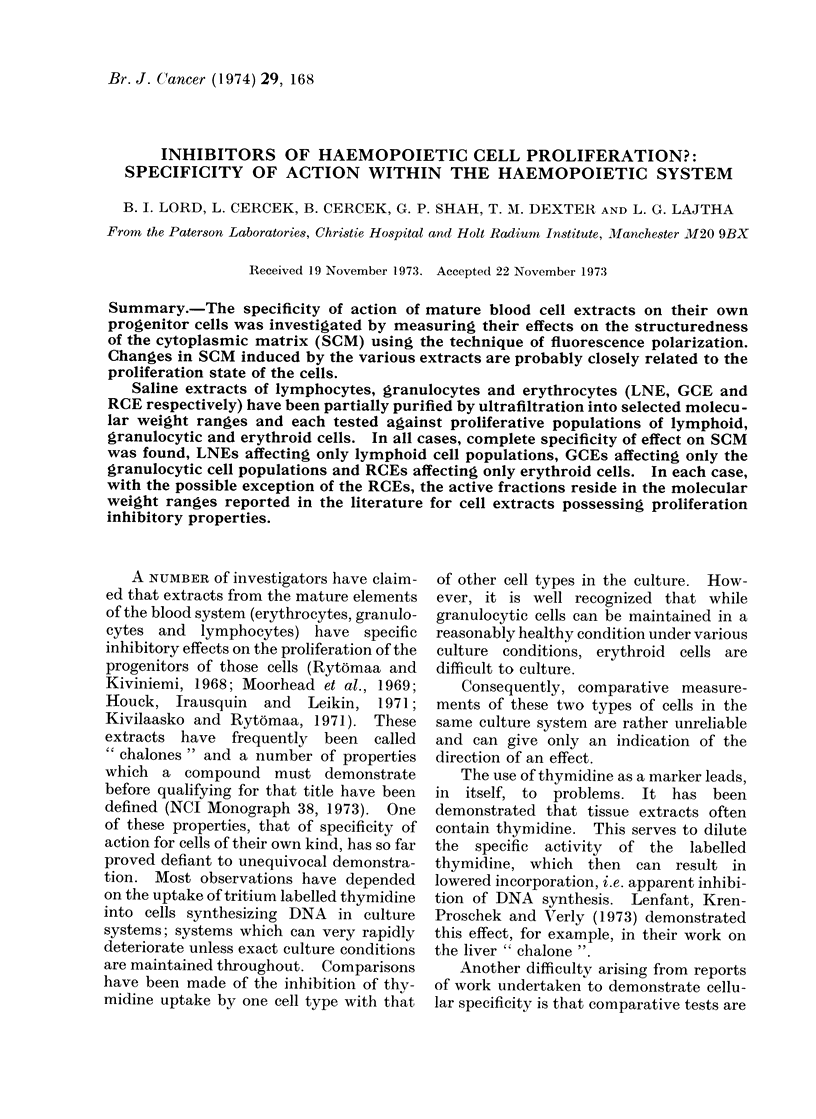

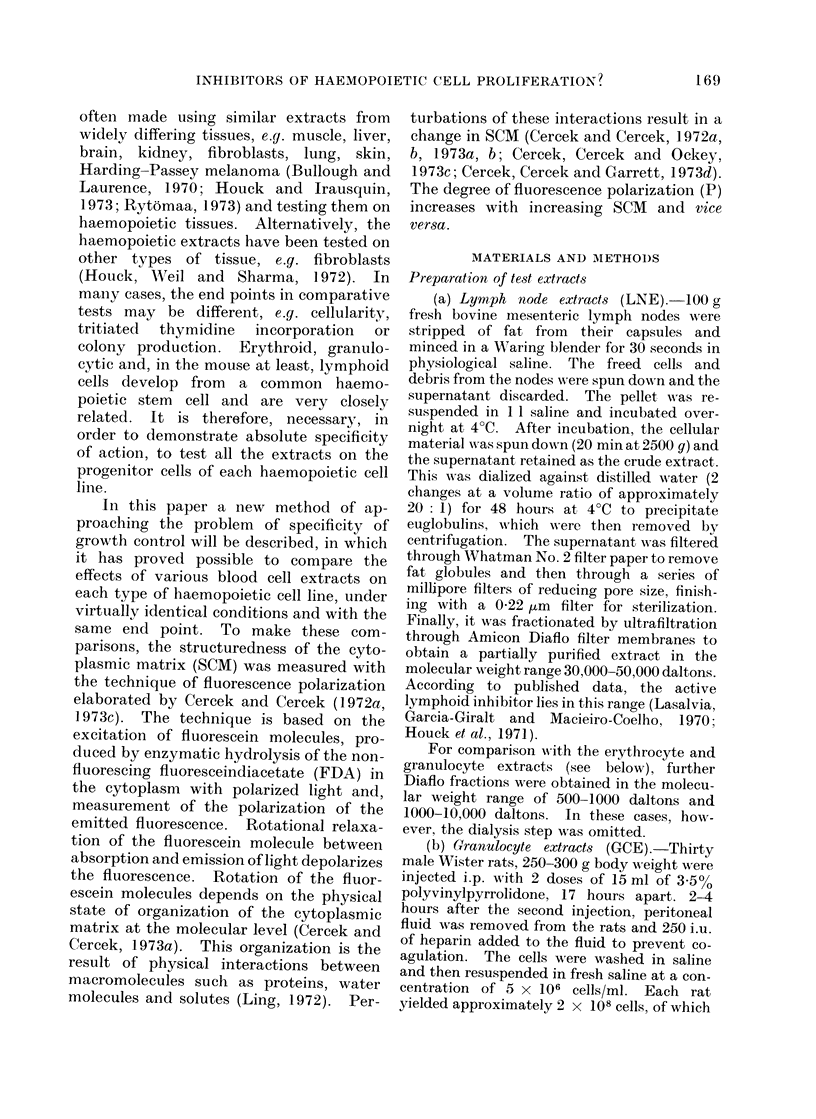

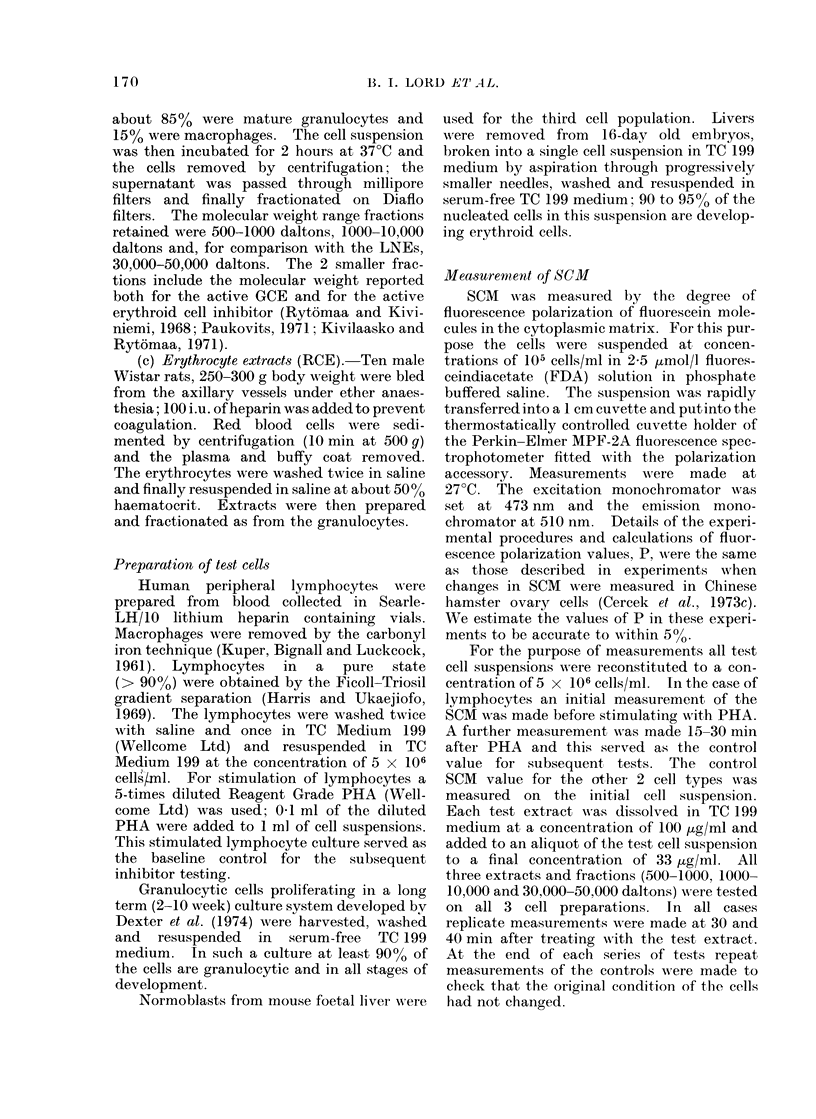

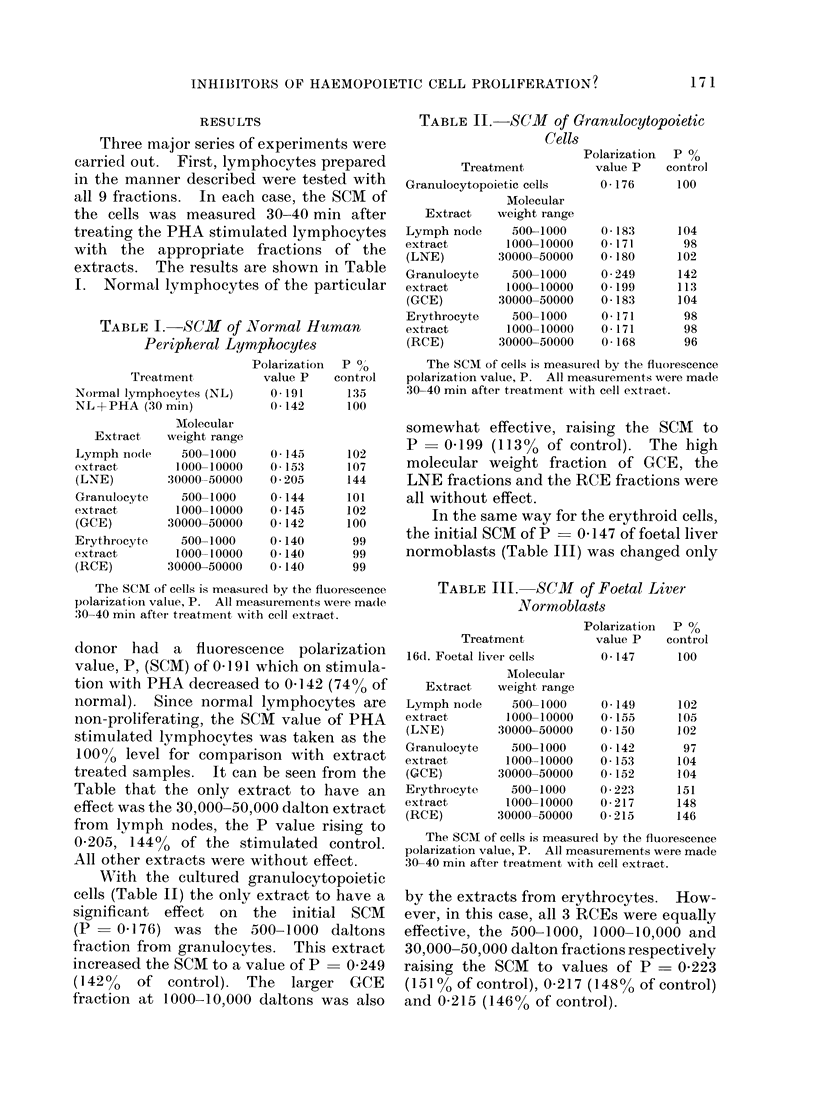

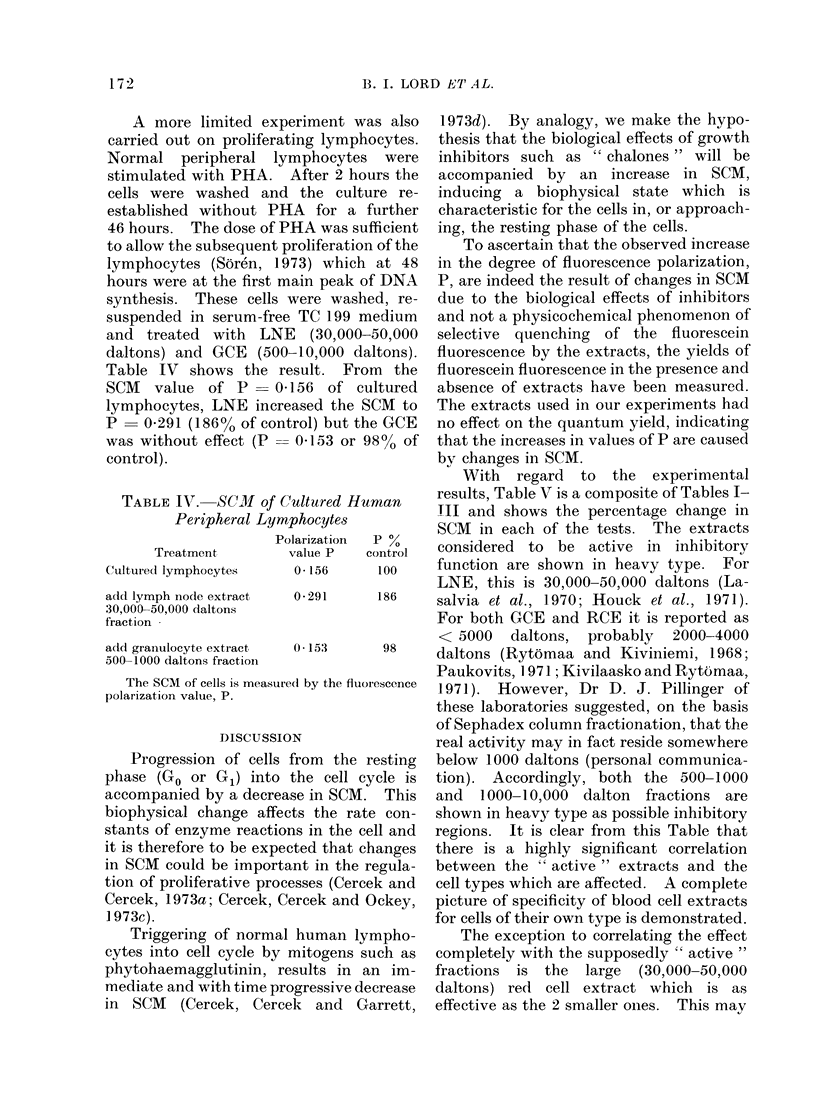

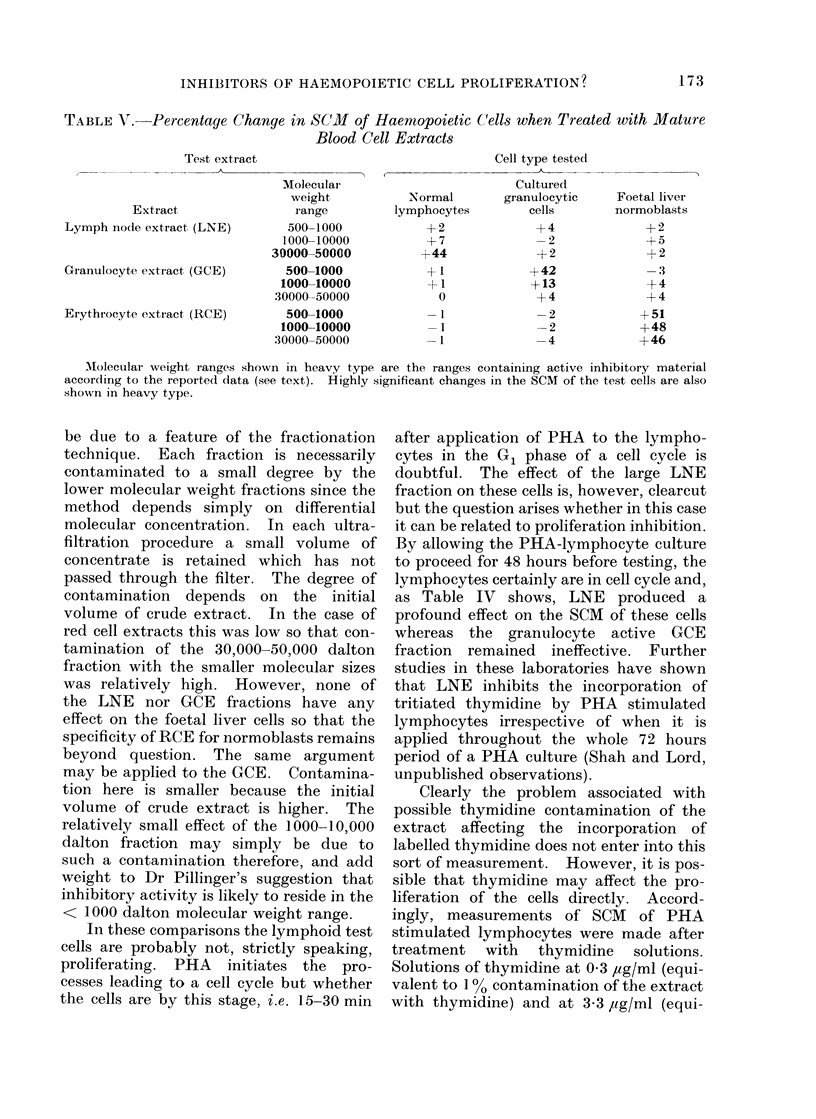

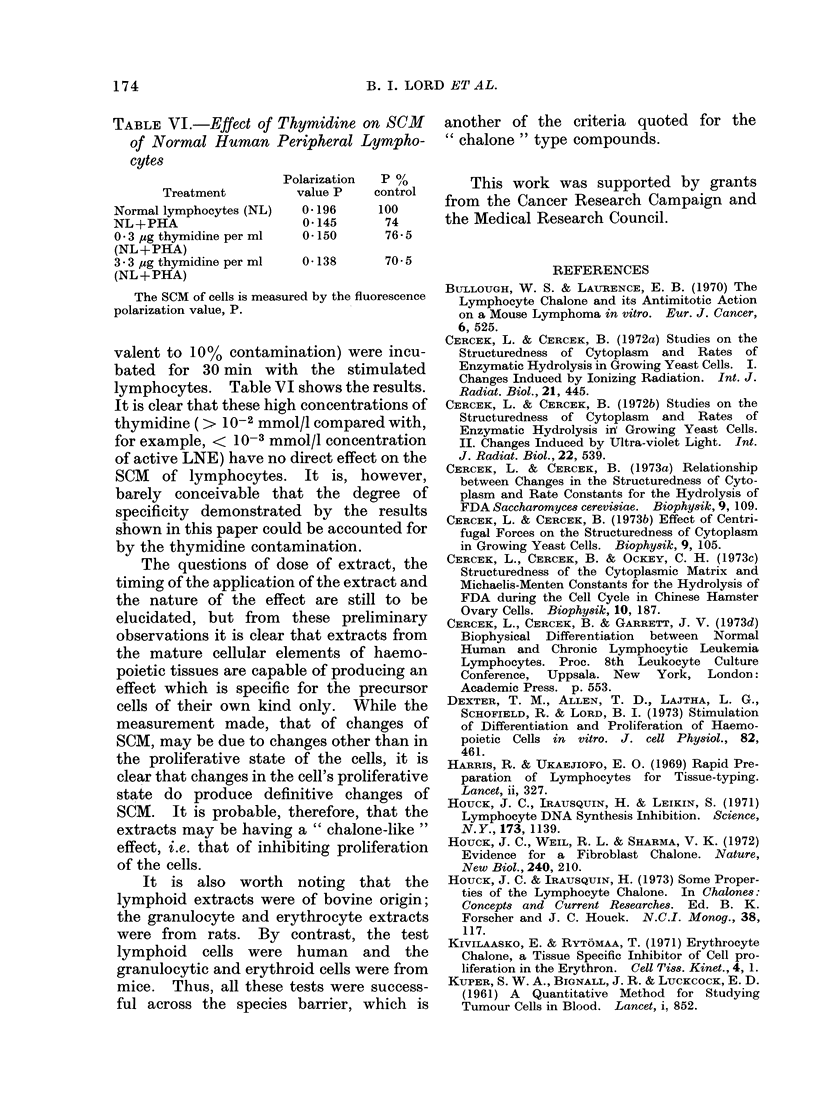

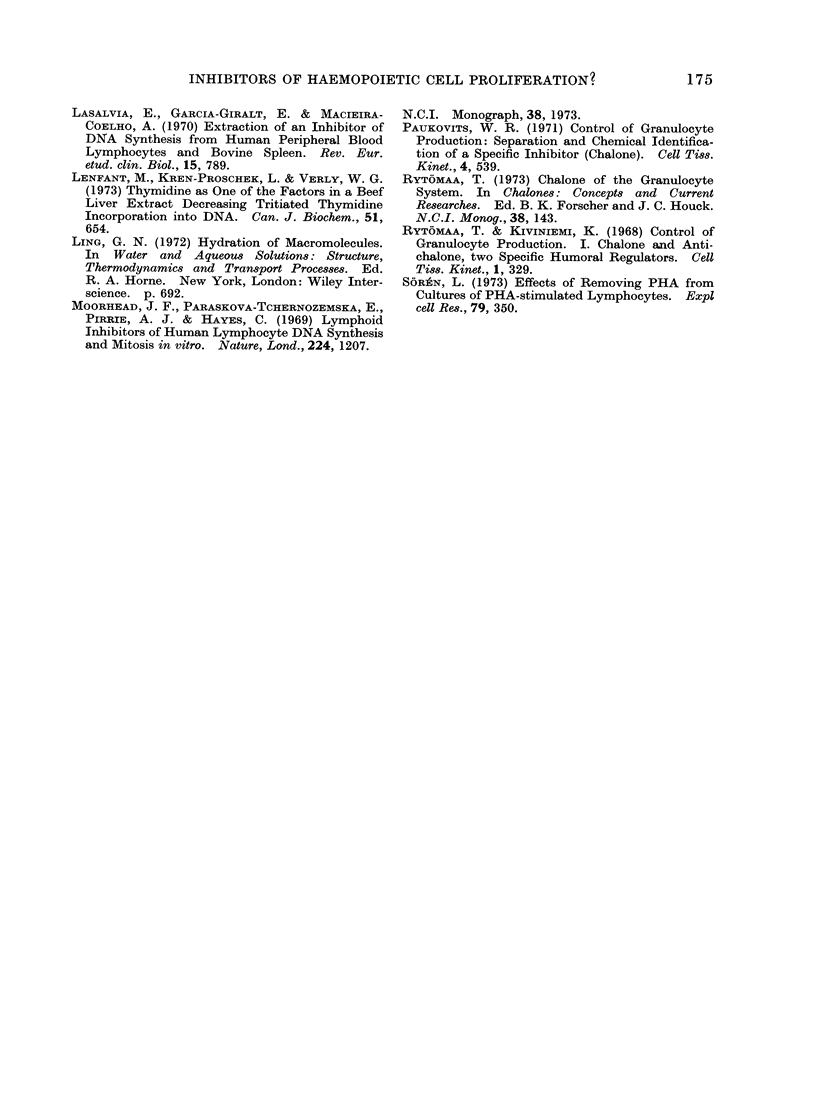


## References

[OCR_00949] Bullough W. S., Laurence E. B. (1970). The lymphocytic chalone and its antimitotic action on a mouse lymphoma in vitro.. Eur J Cancer.

[OCR_00974] Cercek L., Cercek B. (1973). Effect of centrifugal forces on the structuredness of cytoplasm in growing yeast cells.. Biophysik.

[OCR_00979] Cercek L., Cercek B., Ockey C. H. (1973). Structuredness of the cytoplasmic matrix and Michaelis-Menten constants for the hydrolysis of FDA during the cell cycle in Chinese hamster ovary cells.. Biophysik.

[OCR_00969] Cercek L., Cercek B. (1973). Relationship between changes in the structuredness of cytoplasm and rate constants for the hydrolysis of FDA in Saccharomyces cerevisiae.. Biophysik.

[OCR_00955] Cercek L., Cercek B. (1972). Studies on the structuredness of cytoplasm and rates of enzymatic hydrolysis in growing yeast cells. I. Changes induced by ionizing radiation.. Int J Radiat Biol Relat Stud Phys Chem Med.

[OCR_00962] Cercek L., Cercek B. (1972). Studies on the structuredness of cytoplasm and rates of enzymatic hydrolysis in growing yeast cells. II. Changes induced by ultra-violet light.. Int J Radiat Biol Relat Stud Phys Chem Med.

[OCR_00994] Dexter T. M., Allen T. D., Lajtha L. G., Schofield R., Lord B. I. (1973). Stimulation of differentiation and proliferation of haemopoietic cells in vitro.. J Cell Physiol.

[OCR_01001] Harris R., Ukaejiofo E. O. (1969). Rapid preparation of lymphocytes for tissue-typing.. Lancet.

[OCR_01006] Houck J. C., Irausquin H., Leikin S. (1971). Lymphocyte DNA synthesis inhibition.. Science.

[OCR_01011] Houck J. C., Weil R. L., Sharma V. K. (1972). Evidence for a fibroblast chalone.. Nat New Biol.

[OCR_01027] KUPER S. W., BIGNALL J. R., LUCKCOCK E. D. (1961). A quantitative method for studying tumour cells in blood.. Lancet.

[OCR_01023] Kivilaakso E., Rytömaa T. (1971). Erythrocytic chalone, a tissue-specific inhibitor of cell proliferation in the erythron.. Cell Tissue Kinet.

[OCR_01036] Lasalvia E., Garcia-Giralt E., Macieira-Coelho A. (1970). Extraction of an inhibitor of DNA synthesis from human peripheral blood lymphocytes and bovine spleen.. Rev Eur Etud Clin Biol.

[OCR_01041] Lenfant M., Kren-Proschek L., Verly W. G., Dugas H. (1973). Thymidine as one of the factors in a beef liver extract decreasing 3 H-thymidine incorporation into DNA.. Can J Biochem.

[OCR_01055] Moorhead J. F., Paraskova-Tchernozenska E., Pirrie A. J., Hayes C. (1969). Lymphoid inhibitor of human lymphocyte DNA synthesis and mitosis in vitro.. Nature.

[OCR_01063] Paukovits W. R. (1971). Control of granulocyte production: separation and chemical identification of a specific inhibitor (chalone).. Cell Tissue Kinet.

